# Will a trans-articular external fixator placed across the canine stifle allow normal motion if the hinge is centered over the femoral condyle?

**DOI:** 10.3389/fvets.2026.1892017

**Published:** 2026-10-28

**Authors:** Mary A. Baldeon, Simon Roe, David Knazovicky

**Affiliations:** Department of Clinical Sciences, College of Veterinary Medicine, North Carolina State University, Raleigh, NC, United States

**Keywords:** center of rotation, external skeletal fixator, joint immobilization, ligament, range of motion

## Abstract

Traumatic ligamentous stifle injuries are often immobilized with hinged transarticular external skeletal fixation (htaESF), with the hinge placed over the center of the joint. Range of motion (ROM) in htaESF can protect cartilage, however during ROM the stifle center changes. We hypothesized that a hinge placed over the center of the femoral condyles would not allow normal femoro-tibial positioning during ROM. Eight canine pelvic limbs were harvested. Femurs were immobilized and tibias pinned to control rotation during ROM. The femoral condylar center at 135° was marked and lines drawn to a point on the tibia and femur marked on a radio-opaque grid. Radiographs were taken every 5° through ROM. Intersect point (IP) was the intersection of the femoral and tibial lines. The IP x-y coordinates were normalized and plotted. Convex hull area for IP coordinates was calculated. Coefficient variation of the Euclidean distance was calculated. Intersect shift and flexion angle relationship was modeled with a linear mixed model. IP path was non-linear with no consistent pattern between individual legs and within each dog. Convex hull area was large and bilaterally asymmetrical. Linear mixed model analysis revealed a significant inverse relationship between IP shift and stifle angle (*P* < 0.001). The IP of stifle joints during ROM are likely not similar enough to safely execute ROM while in a htaESF. Limitations include previously frozen limbs, and evaluation of only one point in the stifle. Further investigation must be done to determine the ideal construct for ROM without placing abnormal forces on the joint.

## 1 Introduction

Traumatic ligamentous injury in the canine stifle often occurs concurrently with injury to other ligamentous or osseous structures in the stifle (1–3). These injuries are commonly treated with primary repair of the ligaments with or without prosthetic reconstruction, and immobilization of the joint. Immobilization of the joint with a trans-articular external fixator (ESF) after surgical intervention is currently recommended since primary repair provides little strength (3–5). Joint immobilization has been shown to have deleterious effects on the joint leading to cartilage degeneration (6, 7), while controlled joint motion after surgical repair of ligamentous injuries has been shown to be beneficial to both cartilaginous and ligamentous health (6, 8). Indeed, as little as 10? of joint range of motion promotes articular cartilage health (6). It follows that the ideal approach would be a hinged ESF that allows controlled range of motion (ROM) to maintain joint health while also preventing excessive loads on the weak ligament repairs.

A hinged trans-articular external skeletal fixator (htaESF) has been described for the stifle (9–11). An ideally placed hinge restricts motion to the joints' sagittal plane and allows movement around the joints natural axis of rotation (10). The axis of rotation of the stifle has been previously reported to lie caudal to the longitudinal axes of the femur and tibia (12–14). More importantly, the stifle joint is known for its complex motion involving rolling, sliding and rotation resulting in the movement of the center of rotation as the joint moves from flexion to extension (15). This complex motion results in a dynamic axis of rotation of the femur relative to the tibia, and movement of the center of rotation (12, 15, 16). A single axis hinge constrains the axis of rotation of the stifle to a singular point. Because the axis of rotation moves throughout ROM, it is unlikely that a single-axis hinge will allow natural motion of the repaired stifle joint.

The objective of this study was to determine if a single-axis hinged external fixator, with the hinge located over the center of the femoral condyle, will allow some protected motion without forcing the tibia to shift abnormally. This was done by evaluating the location of the intersection of simulated femoral and tibial external fixator bars on lateral radiographs of stifles that were moved from extension to flexion. As the stifle underwent ROM the intersect point (IP) of the simulated external fixator bars was evaluated for shifts between sequential degrees of flexion both in magnitude and direction. A secondary objective was to investigate for a series of sequential angles in which the IP shift was similar. Our hypothesis was that a fixator hinge positioned over the center of the femoral condyles will not allow range of motion while maintaining normal femoral-tibial positioning.

## 2 Materials and methods

### 2.1 Specimen handling

Four canine cadavers euthanized for reasons unrelated to this study were included. Pelvic limbs were grossly evaluated for abnormalities, but not radiographically for pathology. Their pelvic limbs were harvested with the soft tissues of the stifle joint kept intact. The femurs of the harvested limbs were mounted to a frame to immobilize the femur via intramedullary pin (IM). Two perpendicular pins were placed in the tibia, one cranio-caudal and one latero-medial for positioning purposes and to control internal or external rotation of the tibia during range of motion. Three radio-opaque grids were placed, one on the femur secured by two k-wires, one underneath the joint, and one on the tibia also secured by two k-wires (Figure 1). The grids were 3D printed, with each square measuring 1 cm x 1 cm. They were 75% tungsten impregnated polymer to allow for radio-opacity.

**Figure 1:**
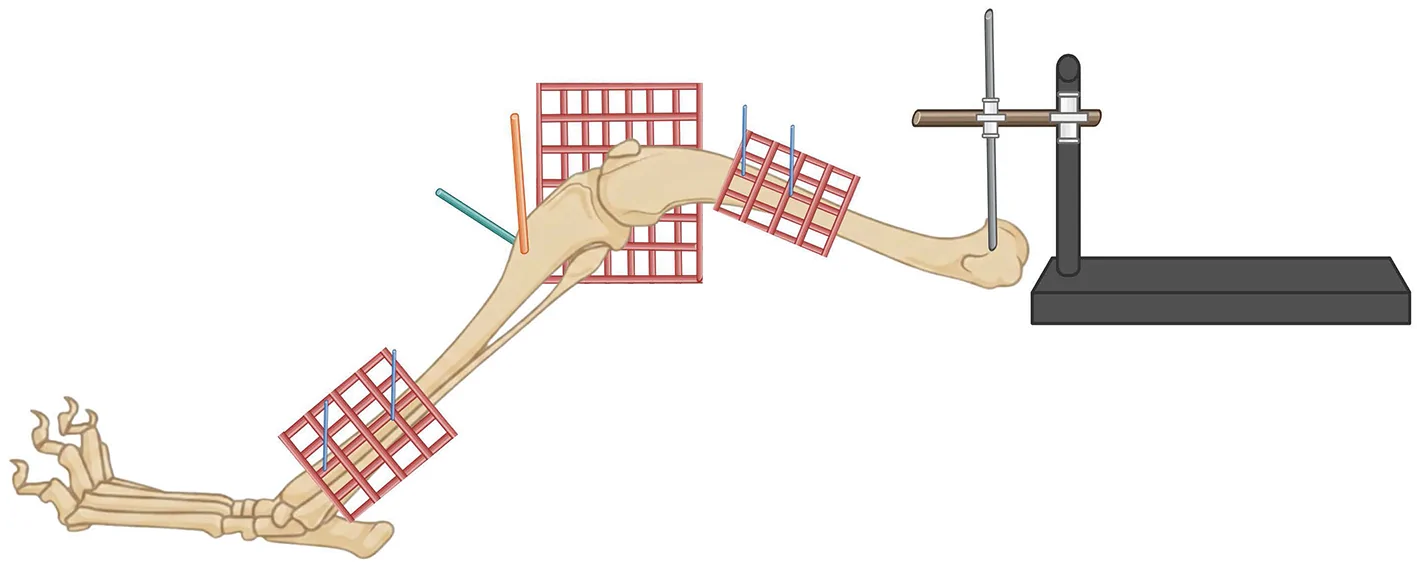
Schematic of radiographic set-up of limbs on an oblique latero-medial view. The weighted stand is represented in black. The brown bar represents an ESF bar connected to the weighted stand and pin. ESF connecting clamps are represented in white outlined with gray. Three Steinmann pins are represented, one latero-medial pin in the proximal femur anchoring the limb in gray, and two in the tibia. The orange pin in the proximal tibia is in the lateromedial direction, and the cyan pin in the cranio-caudal direction. Radio-opaque grids are represented in red. Blue lines represent latero-medial k-wires used to affix grids to the femur and tibia.

At 130° of extension, a point approximating the center of a circle over the lateral femoral condyle was chosen to simulate the hinge point. This point was chosen based on the geometric assumption that a circle is the most likely to have exclusively rotational movement, thus the most likely to demonstrate the least IP shift between sequential angles. To establish the location of the external fixator bars on the femur and tibia, two other points were chosen on the axis of the femur and the tibia, and the location was noted on the grids. Lines were drawn from the hinge point over the femoral condyle to the femoral and tibial points, simulating the optimal location of the connecting bars for that hinge point. The grids were used to ensure identical placement of the simulated ESF bars on the femur and tibia, mimicking the fixed nature of *in-vivo* ESF bars. The stifle was flexed from 130° to 30° with radiographs taken at 5° increments. Using the grids, the x-y coordinates of the IP were recorded. Simulating the fixed ESF allowed evaluation of the natural hinge point of ESF bars if they were unconstrained by a hinge, as the tibia and femur move relative to one another during ROM. All angles were confirmed using a goniometer. The resultant IP x-y coordinates were normalized to the x-y coordinates of the intersection of the simulated bars while the stifle was extended to 130°. The 2-D IP path was visualized by plotting x-y coordinates of each IP sequentially from 135° to 35° for each limb individually. These plots were overlaid by dog for bilateral comparison. This resulted in a self-referenced, dimensionless measurement system ensuring that each IP shift was self-calibrated to each radiograph and avoiding magnification errors inherent in absolute radiographic measurement.

### 2.2 Statistical analysis

All statistical analyses were performed in Python (version 3.12) using the pandas, scipy, pingouin, and scikit-posthocs libraries, with the exception of the linear mixed model which was performed in R (version 4.5.1) using the lme4 and lmerTest packages (17). Statistical significance was set at α = 0.05 for all tests.

To evaluate the variability of the IP path within the same limb across all angles the spatial area covered by each IP trajectory was quantified using convex hull area of the x-y coordinates (18, 19). The convex hull is the smallest convex polygon that encloses all points in a geometric dataset, in this case all recorded IP positions. Convex hull area was compared between individual limbs and between limbs of the same dog.

The Euclidean displacement of the intersect, or intersect shift, for each consecutive degree of flexion for each leg was calculated, along with the standard deviation. The Euclidean displacement equation is $d=\;\sqrt{{{\left(x_{2}-x_{1}\right)}^{2}+\left(y_{2}-y_{1}\right)}^{2}}$, with d representing the calculated Euclidean displacement, and x and y representing the two dimensional coordinates of the intersect point at all sequential flexion angles.

The average intersect shift for all limbs at each consecutive degree of flexion was calculated along with the standard deviation. The coefficient of variation (CV) was calculated for intersect shift across all limbs and flexion angles to characterize the consistency of intersect shift displacement magnitude (20, 21). CV values were defined as low variability < 15%, moderate variability 16–30% and high variability ≥31% (20, 22).

To model the overall relationship between flexion angle and intersect shift across all limbs the intersect was modeled as a linear function of stifle angle using a linear mixed model with a random intercept for each leg defined as specimen number and side (left vs right) (23–25). In addition to the primary fixed effect (stifle angle), a quadratic term (stifle angle^2^) was included in the model to improve model performance, and allow evaluation of the rate of change of IP shift, rather than assuming the same rate for every 5° increment. The model was fit with estimation performed via restricted maximum likelihood estimation and Satterthwaite *t-tests* to test the significance for the intercept and the estimated coefficient (25, 26).

A fixed-effects estimate was calculated for the intercept at the mean-centered angle (angle = 85 degrees), the linear term's coefficient representing the distance change per degree above or below 85 degrees, and the quadratic term's coefficient, which captures the non-linear trend between distance and angle. The angle was mean-centered at 85 degrees to simplify the interpretation of the intercept in the mixed model. (27) Model assumptions were evaluated by examining residual diagnostics. A residuals-vs.-fitted plot was used to check for constant variance, and a Q-Q plot of residuals was used to assess normality.

## 3 Results

### 3.1 Patient demographics

Four dogs, for a total of eight limbs, were included in the study. All limbs had no gross abnormalities, and no abnormalities appreciated radiographically during the course of data collection. Patient information can be found in Table 1.

**Table 1:** Patient demographic information.

Demographic	*n*	Percent of population	Average and standard deviation
Gender			
Male Intact	3	75%	n/a
Male neutered	0		
Female intact	1	25%	
Female neutered	0		
Age (years)			
3	2	50%	
4	0		
5	2	50%	4 ± 1.15
Breed			
Mixed breed	4	100%	
Weight (kg)			
0−10	1	25%	25 ± 14.72
11−20	1	25%	
20−30	0		
30−40	2	50%	

### 3.2 Intersect point path

Coordinate plots of each IP revealed non-linear relationships between each IP at each angle, with no consistent directional pattern across specimens. Indeed, the difference between IP at sequential angles was so markedly different that a line of best fit could not be determined with significance. Spatial paths of all limbs of all dogs are shown in Figure 2 and are separated by dog in Figure 3. Visual inspection of per-dog spatial path plot reveals markedly different trajectories for limbs of the same dog with minimal overlapping. Dog 3 left limb was the most extreme outlier, while Dog 4 was the most contained. The convex hull analysis quantified the spatial area covered by each limb's joint center trajectory, and values for each individual limb are shown in Table 2. The average convex hull area of each limb was 51.22 U^2^ (range 27.28–85.27). The most striking difference is shown in Dog 3 left limb, with a convex hull area greater than 3 times that of Dog 2 right limb. Overall, there is substantial variability between limbs demonstrated by the large range.

**Figure 2:**
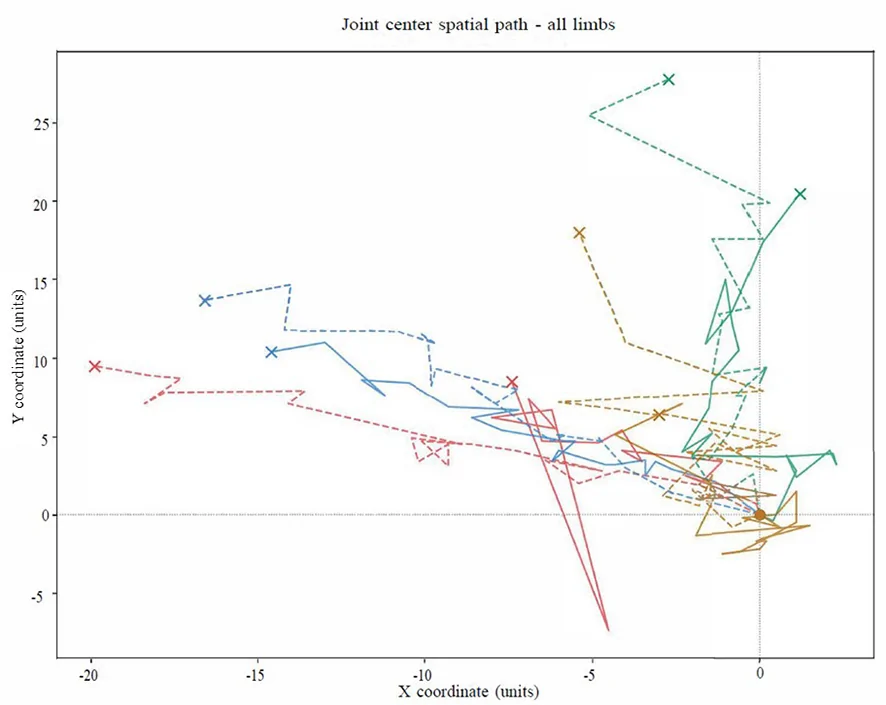
Coordinate plots of all x-y values of limbs of all dogs. Right limbs are in solid lines, and left limbs are in dotted lines.

**Figure 3:**
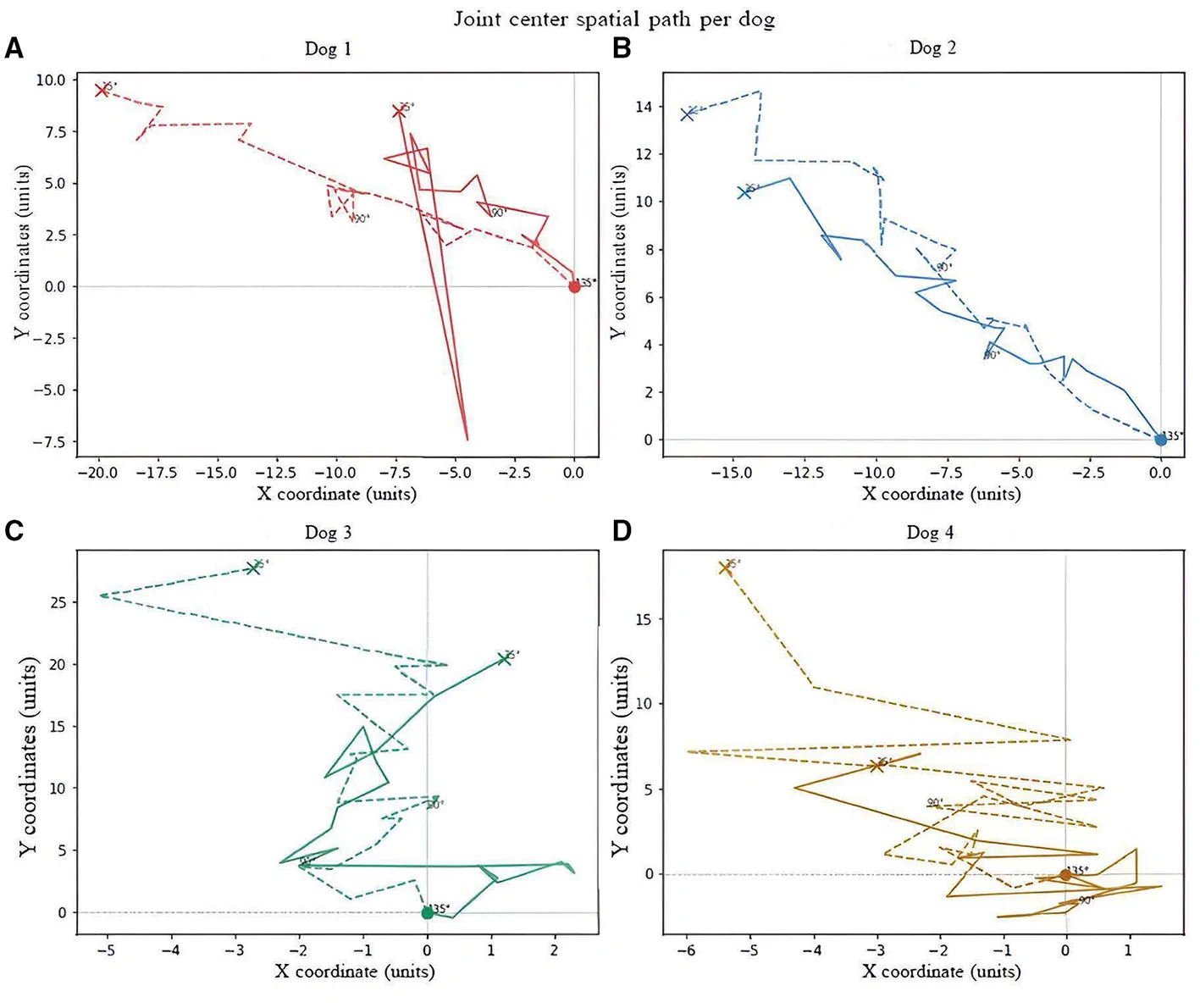
Coordinate plots separated by dog. Right limbs are in solid lines and left in dotted lines. The point at 35° is denoted by an “x” and the point at 130° is denoted by a “•”. **(A)** Dog 1 right and left limb coordinate plots. **(B)** Dog 2 right and left limb coordinate plots. **(C)** Dog 3 right and left limb coordinate plots. **(D)** Dog 4 right and left limb coordinate plots.

**Table 2:** The convex hull area for each limb of each dog and the absolute value of the difference between the two.

Dog	Right limb area (*U*^2^)	Left limb area (*U*^2^)	Absolute difference
1	62.73	41.50	21.23
2	27.28	38.57	11.29
3	57.89	85.27	27.38
4	28.72	67.82	37.10

### 3.3 Limb comparison

The differences in convex hull area for both limbs of the same dog were calculated to evaluate symmetry in spatial trajectory, shown in Figure 4. These values are shown in Table 2. Bilateral asymmetry was present in all four specimens with differences between right and left limbs ranging from 11.3 to 39.1 U^2^. In order to evaluate any overlap of the path of the IP, the mean x, y position of that limb's IP path was plotted with a 95% confidence ellipse. Dog 1 shows a large horizontal separation between left and right with minimal overlap of ellipses. Dog 1 left ellipse is shifted to the right, indicating that the left leg IP path migrates further laterally. Dog 1 right ellipse is larger, reflecting the high variability and erratic path. Dog 2 shows separation between right and left, albeit smaller in magnitude than Dog 1. These ellipses overlap, and were the most similar for all dogs, despite differences in size of ellipse. Dog 3 demonstrates means that are the closest together out of all dogs in X, but are separated in Y. The ellipses overlap, however the difference in size indicates lack of symmetry. Dog 4 demonstrates minimally overlapping ellipses, despite being the most spatially contained limb, reflected in its convex hull area which was the smallest of the 4.

**Figure 4:**
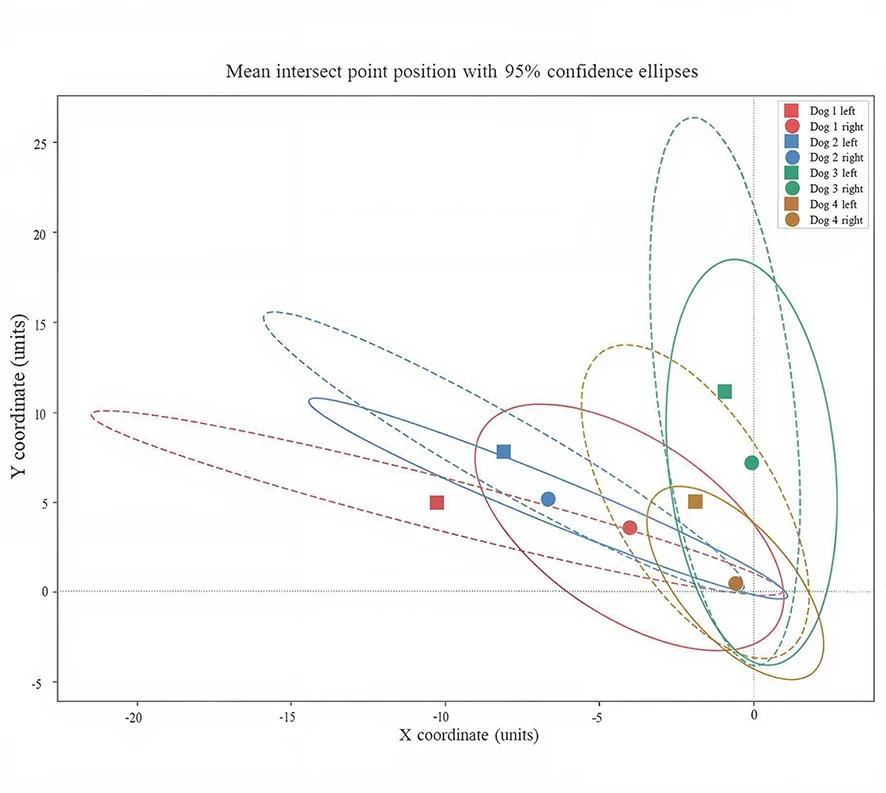
Graphical representation of the mean x-y position of that limb's IP averaged across all flexion angles. Right legs are shown and circles with a solid 95% confidence interval ellipse, and left legs in squares with dotted 95% confidence interval ellipse.

### 3.4 Intersect point shift

The average distance between each intersect point (IP) for each angle overall and in the right (R) and left (L) stifles of all dogs is shown in Figures 5, 6 respectively. There is an overall trend of the IP shift decreasing as the angle of the stifle increases. No angles had a calculated CV of < 15%, and only 4 angles had a calculated CV < 30% shown in Figure 7. Of the angles with a CV < 30% 2 were found in left limbs, and 2 in right limbs, none at the same angle between legs, and none in sequential angles. All other angles fell within a CV greater than 30%, indicating high variability.

**Figure 5:**
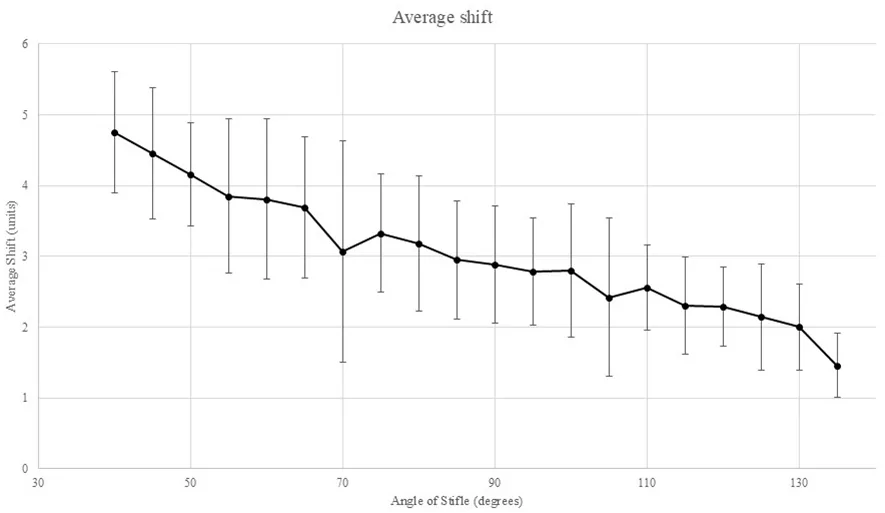
The average shift between intersect points across all limbs. The black line with dots represents the average value at each respective angle. The black bars are the standard deviation.

**Figure 6:**
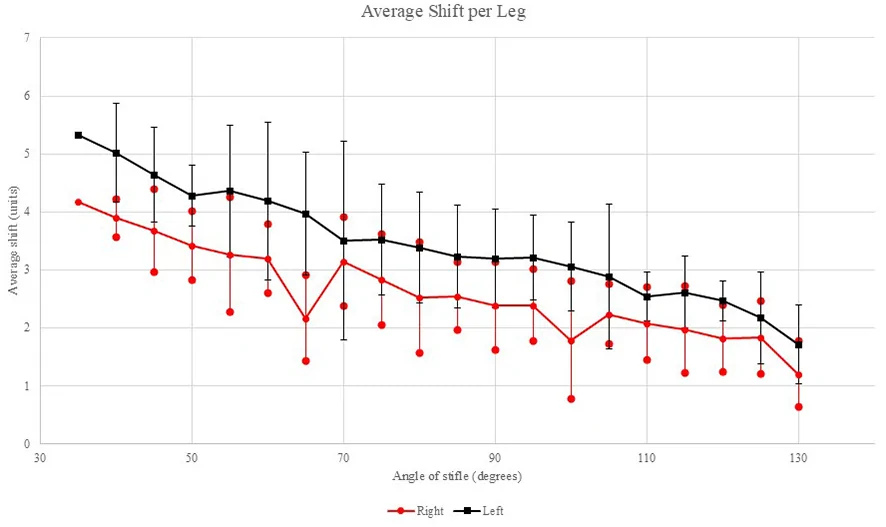
The average shift between intersect points of left and right limbs. Left is shown in black, right in red. Standard deviation for the left limb are black bars with line end caps, standard deviation bars for the right limb are red with round end caps.

**Figure 7:**
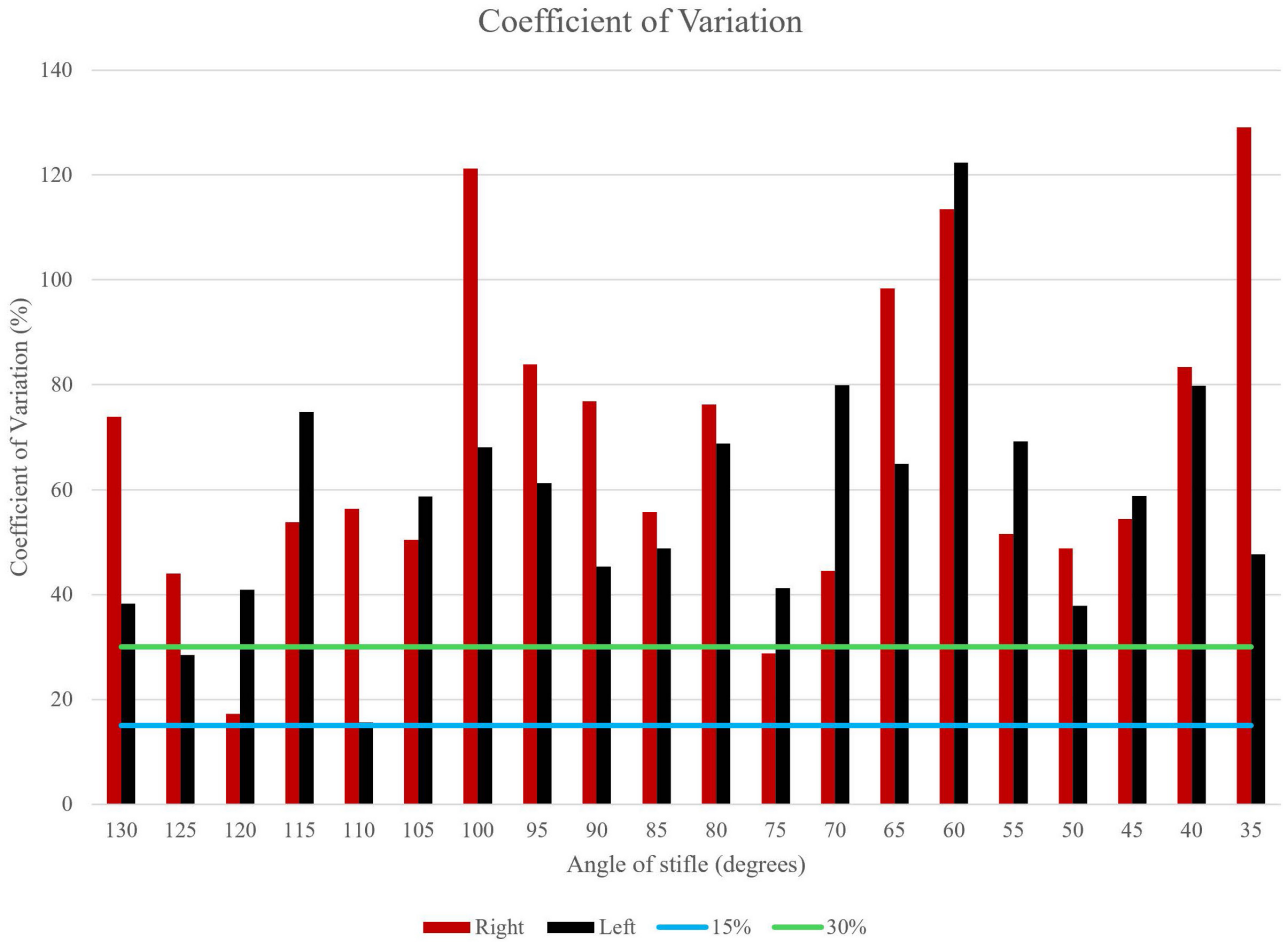
This graph shows the CV at each angle of the stifle. Left limbs are represented in black, and right in red. The green horizontal line indicates a CV of 30%, which is the cutoff for moderate variability. The blue horizontal line indicates a CV of 15%, which is the cutoff for low variability.

### 3.5 Linear mixed model

A scatter plot of the linear mixed model is shown in Figure 8. The linear mixed model revealed a significant negative correlation. A statistically significant inverse relationship between the angle of the stifle and the intersect shift was found (*p* < 0.001). When the angle of the stifle changes by 1° relative to the mean angle degree at 85, the absolute distance (irrespective of direction) between points decreases by 0.027 units (primary linear term), with a small but statistically significant quadratic term's effect (0.000038 per degree^2^). The significant quadratic term indicates the distance of IP shift decreases slower at a larger stifle angle which capturing a concave-upward relationship between the distance and angle rather than a strictly linear trend. The fixed effects estimates are shown in Table 3.

**Figure 8:**
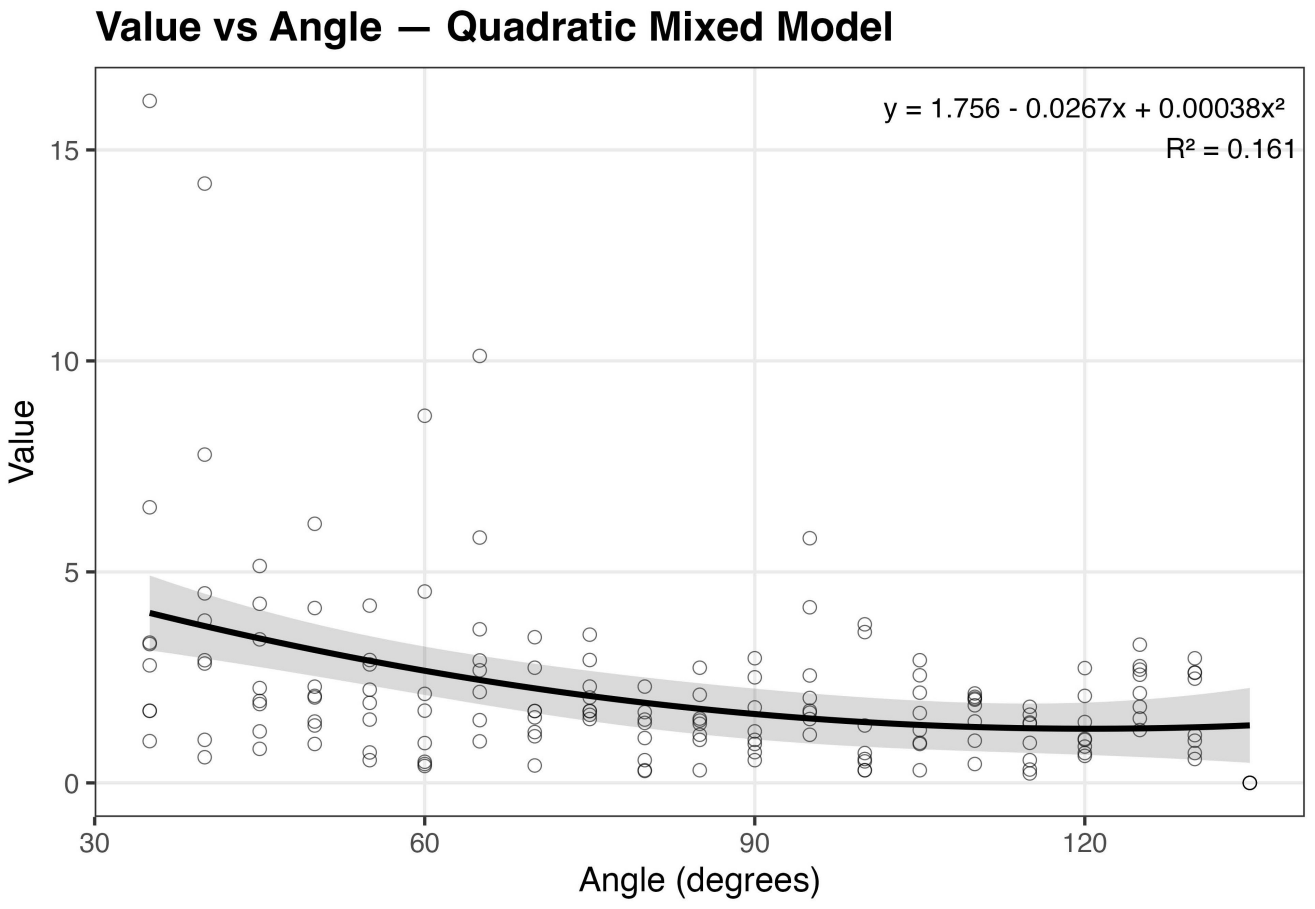
A graphical representation of a linear mixed model exploring the association between the calculated intersect shift and the angle change. The x axis is the degree of flexion from 35–135 and the y-axis is the magnitude of the intersect shift. The black solid line represents the estimated linear function between value and angle, and the gray shaded area is the 95% confidence interval for the estimated line. The circles represent each individual value.

**Table 3:** Fixed effects estimate.

Estimate	Estimate Value	Standard error of the estimate value	Degrees of freedom	*T* value	*p*-value
Intercept (Angle = 85 degree)	1.756	0.308	13	5.696	*P* < 0.01
Linear term	−0.0267	0.00479	158	−5.568	*P* < 0.01
Quadratic term	0.000378	0.000177	158	2.119	0.036

Estimate shows the estimated intercept, and estimated coefficients for linear term and quadratic term. The negative sign indicates the inverse relationship between intersect shift and angle.

## 4 Discussion

Our findings cause us to accept our hypothesis, that the path of the IP is likely too mobile to be able to safely perform stifle ROM when a trans-articular ESF with a single-axis hinge is positioned over the center of the femoral condyles without placing abnormal forces on the joint. Coordinate plots of the IP throughout range of motion are dissimilar between dogs and limbs of the same dog. Every limb shows direction reversals, backtracking and erratic steps. The inter-dog variability is substantial, with paths of each limb occupying entirely different spatial regions between dogs. When evaluating bilateral symmetry, no dog demonstrates similar paths through space. While overall trends show movement of the intersect shift caudally as the stifle extends in both this report and the literature (28), the paths through space show no similarity. There are no IP shifts between consecutive angles that are similar enough to ensure normal forces are exerted on the joint during ROM. While 10° of movement is the minimum for maintenance of joint health (6), even 5° of movement results in marked changes in the position of the IP.

To accurately compare IP paths between dogs and limbs, all x-y coordinates were normalized to zero. It could be argued that anatomic and conformational differences contributed to the significant differences in limb paths, however, the absence of similarity between limbs of the same dog indicates that anatomy may play a smaller role in IP trajectory. Only four dogs were included, an objectively small number, yet the degree of variation of IP path was significant. When extrapolated to the dog population as a whole, it is reasonable to suggest that this trend can be expected between dogs, and between the limbs of the same dog.

Convex hull analysis further supports our hypothesis. By quantifying the spatial area covered by the IP trajectory, we were able to evaluate how much 2D space the IP covers across the full ROM of the stifle. This revealed a massive range of high magnitude values indicating the IP wanders over a large spatial area rather than staying in a tight cluster. Even when evaluating a mean IP shift, a calculation most likely to lose significance of data, neither mean IP shift nor 95% confidence ellipse occupied similar regions between legs. Even dogs with means that are close together or tightly contained 95% confidence ellipses showed barely similar IP paths. All means are in different regions of the plot, indicating extreme inter-dog variability. Symmetrical IP paths would show an overlap of the calculated means for each limb (circle and square). Trajectory patterns between dogs also differ, with Dog 1 and 2 migrating more in cranial and caudal directions, and Dogs 3 and 4 migrating more in proximal to distal directions.

Plots of the magnitude of IP shift averaging all dogs, and per leg demonstrate large standard deviations indicating variability of the magnitude of IP shift for each sequential angle of the stifle. CV analysis supports this unpredictable IP shift, with 90% of angles falling outside of the moderate variability cutoff, indicating that IP displacement is substantially inconsistent across range of motion regardless of specimen or limb. It should be noted that there were no angles of the stifle below the moderate variability cutoff that were sequential, further supporting that there is no range of angles with minimal IP displacement. The linear mixed model demonstrates this at a population level, showing that on average displacement decreases with angle, which is supported by the current literature (28). While the distance between displacement for every 1° increase in flexion angle is modest, the remaining analyses show that there is significant individual variability.

While the findings of this study do not support performing ROM in a stifle immobilized by htaESF, the clinical problem remains that ROM is vital for joint health. Despite findings in the human literature that a mono-axial htaESF both stabilizes the human knee and allows physiologic knee movement in a limited range of ROM (29), based on our data, the same likely cannot be said for dogs as no sequential IP locations were similar. Not only could the abnormal forces being placed on the joint increase strain of the ligamentous repair, they may place abnormal forces on the ESF construct, which could result in premature construct failure (10).

An argument can be made that clinical patients undergo ROM in an htaESF and proceed to heal their ligaments. While this may indicate that regardless of our findings ROM can be performed, there are no studies to date investigating if the phenomenon reported here causes construct or surgical complications. This study sought to address that paucity by demonstrating that the intersect point of htaESF bars as the femur and tibia move erratically relative to one another during ROM. It may be that the material properties of the construct, the elasticity of the surrounding tissues, or the tolerance of individual dogs play a role in the positive outcomes of htaESF reported in the literature (3, 9, 10). The type of stifle injury may also play affect tissue tolerance of the forces from drastic IP shifts. Presence or absence of intact patellar, collateral, or cruciate ligaments may influence the ability for patients to heal despite the findings in this study.

It remains unknown how much IP shift is tolerated *in-vivo*. Construct complications are common in ESF (11). One study investigating htaESF in stifles reported construct complication rate in 3 out of 10 dogs, and medial collateral ligament repair complications in 5/10 dogs (9). Jaeger et al. reported a construct complication of 5/14 animals, however that study also evaluated htaESF in the tarsus (10). It is reasonable to posit that the constraint of a hinge may provide a cantilever anchor at the hinge *in-vivo*. As the stifle goes through ROM and the hinge constrains the ESF bars, abnormal stresses in the frame, in the peri-articular tissues or within the joint may occur. It also remains unknown if there is a magnitude or directionality of IP shift that is tolerated in dogs. Further research into the effect of these abnormal forces is warranted. The results of the linear mixed model reveal that at higher angles of extension IP displacement magnitude decreases at a slower rate, indicating that there may be a range of angles between 85°-135° that may be tolerated. However, without the proof that ROM at these angles does not exert abnormal forces, and in the light of the grossly erratic intersect point path shown, more work is needed to determine if a safe range of angles exists.

The solution to this issue is not straightforward. One could place a triangular ESF and remove the caudal spanning bar and the clamps between the femoral and tibial connecting bars in order to perform non-load bearing ROM. However, unsupported non-load bearing ROM of a ligamentous repair has been shown to increase reconstruction failure rates in humans (3, 30). Previous studies have shown that performing ROM in a htaESF results in sustained joint motion and earlier return to weight bearing, however, complications involving the construct were observed in 5 out of the 9 patients in that study (10).

This study has limitations. While the limbs were grossly evaluated for defects, deformities and pre-existing conditions, radiographic screening prior to experimentation was not pursued. No abnormalities were noted grossly, and none were found radiographically despite absence of an orthogonal view. To the authors knowledge, this particular methodology has not been reported. However, this closely approximates *in-vivo* htaESF, by using the same points on the axes of the femur and tibia to evaluate where the intersect point, or proverbial hinge, should ideally be at that degree of flexion or extension. Only one location of the hinge was evaluated in this study, and therefore the present results should not be interpreted as evidence against all possible single-axis hinged trans-articular fixation strategies. The center of the femoral condyles was chosen due to its anatomic similarity to a geometric circle, which was considered most likely to allow pure rotation. This point also approximates the general location of the Instantaneous Center of Rotation of the canine stifle with intact cruciate ligament (16). It logically follows that a geometric circle may reveal a series of angles at which the IP shift is reasonably similar, which this study demonstrated to be false. It could be argued that controlling tibial torsion could have affected the tibial “bar” and thus the IP, however, an *in-vivo* htaESF would also control tibial torsion. As the goal was to approximate *in-vivo* htaESF it is unlikely that tibial torsion control affected these results. The medio-lateral shift of the IP was not evaluated in this study, since it is not relevant to the behavior of an htaESF. In addition, as with other geometric methods used to characterize joint motion, small errors in point placement or angular measurement may be amplified in the calculated intersect-point location, particularly when sequential motion increments are small. The limbs were frozen, which may have affected soft tissue behavior during ROM. At this time, it is unknown if muscles of a live patient exert forces on the stifle during passive ROM that could affect these findings. Finally, while divergence between a fixed hinge axis and the native motion pathway may theoretically generate abnormal joint loading, forces were not measured in the present study.

In conclusion, based on the results of this study, a htaESF with the hinge located over the center of the femoral condyles will likely lead to erratic movement of the tibia relative to the femur as the joint is moved through a range of motion. A solution to this problem is yet to be discovered. However, with the advent of 3D printing, it is not unreasonable to posit that a hinge could be designed that allows for more degrees of freedom while still controlling movement of the stifle during ROM. An alternative method of immobilization, or alternative placement of the hinge location may allow safe ROM, and warrants further investigation. Overall, these results indicate that performing ROM on a ligamentous repair in a stifle stabilized with a htaESF with the hinge located over the center of the femoral condyles may result in abnormal forces not only on the ligamentous repair, but on the htaESF as well.

## Data Availability

The raw data supporting the conclusions of this article will be made available by the authors, without undue reservation.
